# Quality parameters of natural phenolics and its impact on physicochemical, microbiological, and sensory quality attributes of probiotic stirred yogurt during the storage

**DOI:** 10.1016/j.fochx.2022.100332

**Published:** 2022-05-18

**Authors:** Anuradha Wijesekara, Viraj Weerasingha, Shishanthi Jayarathna, Hasitha Priyashantha

**Affiliations:** a Department of Animal & Food Sciences, Faculty of Agriculture, Rajarata University of Sri Lanka, Anuradhapura, Sri Lanka; b Department of Molecular Sciences, Swedish University of Agricultural Sciences, Box 7015, Uppsala SE 750 07, Sweden

**Keywords:** Plant pigment, Fermented milk, Color stability, Natural colorant, colored stirred yogurt

## Abstract

Physicochemical, microbiological, and organoleptic properties were evaluated for probiotic stirred yogurts with plant pigments; 10% Hibiscus, 4% Turmeric, 6% Spinach, and 4% Blue pea, over 14 days at 4℃ compared to the colorless control. The color of yogurts were stable without sedimentation or adverse effect on physicochemical or sensory properties, although an increase of L* value observed over the storage. The microbial analysis confirmed the viability of probiotics (>9 logs CFU/mL) in all yogurts over the storage. Turmeric added yogurt resulted in the highest b* value, total phenolic content (72.6 mg GAE/L) and sensory score for color, while spinach added yogurt ranked the lowest in flavor at the end of storage. Results demonstrated the color stability of studied plant pigments in stirred yogurt with varying physicochemical and sensory properties. Addition of natural colorant in yogurt is recommended. Improved methods for extracting pigments and their health effects should be further examined.

## Highlights

• Four types of plant-derived pigments were assessed in stirred yogurt production.
• Yogurts were stable without sedimentation or noticeable decolouration.
• Plant pigment addition did not exert adverse effect on the survival of probiotics.
• Turmeric addition resulted in the highest sensory acceptance, b* value and total phenolic content.
• The use of plant pigments in stirred yogurt production is technologically feasible.

## Introduction

Color is a noteworthy sensory attribute in food selection. The addition of food colorants enhances or alters the food color that would otherwise lose due to changes in the atmosphere, temperature, or light (Amchova, Kotolova, & Ruda-Kucerova, 2015). The color of the food is closely associated with several quality parameters, such as freshness, desirability, ripeness, and safety (Toldra & Nollet, 2021). At present, there is a growing trend of replacing synthetic food colorants with natural pigments with advances in food processing and preservation. There are several adverse health effects of synthetic food colorants; tartrazine E102, quinoline yellow WS E104, sunset yellow EFSF E110, carmoisine.

E122, ponceau 4R E124, and Allura red AC E129 are associated with the attention of deficit hyperactivity disorder in children and allergic reactions (Luzardo-Ocampo, Ramirez-Jimenez, Yanez, Mojica, & Luna-Vital, 2021). Thus, the usage of natural colorants, e.g. pigments, originating from plants, animals, microbes, or minerals in several food products has been investigated. Anthocyanin (berry fruit juice, carrot, red cabbage, hibiscus), carotenoids (curcumin, b-carotene from alpha-alpha), betalains from beet, and carminic acid from the cochineal insect is some commercially exploited and approved natural colorants in the United States (Luzardo-Ocampo et al., 2021).

The use of natural colorants based on plant pigments in dairy products is challenging due to their potential impact on the technological and functional properties of the final product. A variety of plant-derived colorants have been tested on the color of the yogurt, e.g. betalains of Ayrampo (O*puntia soehrensii*) seeds has shown to decrease the L* (lightness), curcumin incorporation exhibited a decrease of a* (red/green), and b* (yellow/blue) values with time (Luzardo-Ocampo et al., 2021). Grapes’ anthocyanin in Kefir has been reported to lower the pH, L*, and a*, however, it consists of similar physical properties as natural kefir without colorants (Montibeller, de Lima, Tupuna Yerovi, Rios, & Manfroi, 2018). Yet, the total replacement of artificial colorants with natural pigments challenges the color stability of the product. Galaffu, Bortlik, and Michel (2015) showed natural pigments have shown poor stability against pH, light, oxygen, heat, and deleterious enzymes.

*Hibiscus rosa-sinensis L*. (Hibiscus) which is commonly available in Sri Lanka contains anthocyanin with the main compound cyanidin-3-sophoroside (Nakamura, Hidaka, Masaki, Seto, & Uozumi, 1990). In addition, *Clitoria ternatea L*. (Blue pea) consists of delphinidin anthocyanins is responsible for its deep blue colour (Campbell, Marble, & Pearson, 2019). Luzardo-Ocampo et al. (2021) reported that anthocyanins are water-soluble polyphenolic pigments of the flavonoid group, which delivers a range of colors from orange to blue-purple. Chlorophyll pigments are widely available in green vegetables and fruits with the potentials to be used as a food colorant. Hence, *Spinacia oleracea* (spinach) chlorophyll pigments are a potent green colorant for yogurts. *Curcuma longa* (Turmeric) with the pigment curcumin has been used for centuries as a food preservative and a spice (Popuri & Pagala, 2013) as well as a natural colorant in food (El-Shazly, El-Tawil, & Hammam, 2011). Plant-derived pigments possess nutraceutical properties, such as antioxidant, antifungal, antibacterial, anticarcinogenic, and anti-inflammatory properties (El-Sayed, 2020, Lakshan et al., 2019, Popuri and Pagala, 2013). Therefore, the incorporation of plant-derived colorants might deliver value-added benefits to the consumers, apart from their coloring effect. However, in the literature, studies that compare the use of aqueous extracts of hibiscus, blue pea, spinach, and turmeric in probiotic stirred yogurt are scarce to our knowledge. Hence, the study hypothesized that the use of natural plant pigments of hibiscus, blue pea, spinach, and turmeric into the probiotic stirred yogurts will have color stability throughout the storage without adverse impacts on the physicochemical and sensory properties of the yogurt.

## Materials and methods

### Collection of milk, natural pigment sources, and their extraction

Fresh whole bovine milk was obtained from the Faculty Farm, Rajarata University, Puliyankulama, Sri Lanka. Fresh flowers and spinach leaves were collected from a home garden in Anuradhapura, Sri Lanka, while turmeric rhizomes were bought from a retail shop in Matale, Sri Lanka.

### Preparations of plant aqueous extracts

#### Anthocyanin from hibiscus

Anthocyanin aqueous extraction was prepared according to Sindi, Marshall, and Morgan (2014) with slight modifications. Hibiscus petals were washed in running water and sun-dried. Afterwards, those were oven-dried (YCO-010, Taiwan) at 50℃ for 24 h (Iwalokun & Shittu, 2007). Dried hibiscus petals were boiled in water (solvent) for 10 min. The final extract was obtained by filtering using a cotton filter.

#### Curcumin from turmeric

Aqueous extraction of curcumin was prepared according to Shalaby and Amin (2018) with brief modifications. Turmeric rhizomes were washed in flowing water to clean the impurities, direct dried and oven-dried (YCO-010, Taiwan) at 105℃ for 3 h (Sahne, Mohammadi, Najafpour, & Moghadamnia, 2016). Grinding was done using a sterile electric blender (CML-7360065-Japan) and it was practiced after cutting turmeric into small pieces to increase the surface area. Next, sieved powder (1 mm mesh size sieve) was boiled in distilled water (1:10; w/v) and let to precipitate completely. Thereafter, the supernatant was filtered using a cotton cloth to collect a clear solution. Then the aqueous extract of curcumin was heated at 80℃ for 20 min in a water bath (YCW-010E, Gemmyco) and cooled immediately in an ice bath until the temperature reached 5℃.

#### Chlorophyll from spinach

Spinach leaves were pasteurized at 75℃ for 4 min (Hot Air Sterilizer, YCO-010, Taiwan) (Aamir, Ovissipour, Rasco, Tang, & Sablani, 2014) and the aqueous extraction was followed according to Iriyama, Ogura, and Takamiya (1974) with slight modifications. Spinach leaves were homogenized for 3 min with 5% v/v ethanol (Food Chemical Codex, 2017) to a ratio of 1:5 (w/v), using an electric blender. The obtained green juice was filtered through a pad of cotton to remove any coarse debris.

#### Anthocyanin from blue pea

Undamaged, disease-free, fully bloomed blue pea flowers were oven-dried at 50℃ for 24 h after washing with running water (Lakshan et al., 2019). The aqueous extraction was carried out as per Marsin et al. (2020). Dried blue pea flowers were weighed at a 1:20 ratio (g/mL) to distilled water. The mixture was subjected to microwave extraction at 60% power using a domestic microwave (LG, Korea) oven at a 1 min extraction time. Then, the extract was filtered using a cotton pad. The filtered extract was centrifuged at 822× g for 30 min to remove the precipitant and the extract was used.

#### Preparation of colored probiotic stirred yogurt

Colored probiotic stirred yogurt was manufactured using fresh bovine milk. Filtered milk was preheated (50–60 °C for 5 min) and homogenized for 2 min (HBB250, Hamilton Beach, Virginia). Milk powder (2%), and sugar (8%) were incorporated and pasteurized up to 90–95 °C for 5–10 min.

**Fig. 1:**
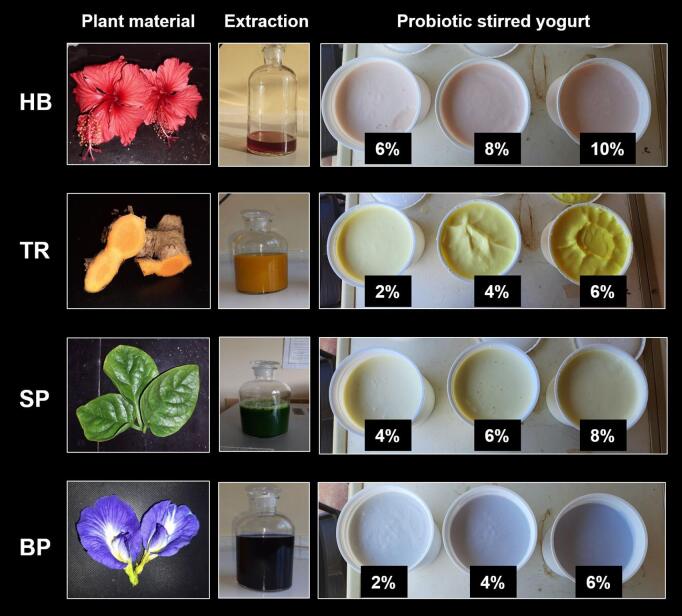
Probiotic stirred yogurt with varying color concentrations; HB: Hibiscus, TR: Turmeric, SP: Spinach, BP: Blue pea.

After, cooling to 44 °C, inoculation of 0.004% of starter cultures (*Streptococcus thermophilus and Lactobacillus delbrueckii* subsp*. Bulgaricus,*) (YF-L812, Chr-Hansen, Denmark) along with 0.004% of the probiotic strain *Bifidobacterium animlis* subsp. *lactis* (Bb-12) (nu-trish® LGG®, Chr Hansen, Denmark) was inoculated. Then, incubated (IN30, Memmert, Germany) at 44 °C for 7–8 h until the pH reached 4.6. Based on a preliminary sensory study (Fig. 1) best concentration of color for each plant-derived colorant was assessed to use in this study. The formed yogurt was stirred using a hand beater (Philips HR1456, China) with fresh raw cow milk at a ratio of 3.125 mL:100 mL along with suitable concentrations of natural colors for 20 s after cooling the incubated yogurts for one night. Incorporation of varying levels of plant extracts (i.e., Hibiscus, Turmeric, Spinach, Blue pea) resulted in yogurts with varying colors and their intensities are shown in Fig. 1. Five types of probiotic yogurts were prepared by varying plant colorants, e.g. control (C), without any plant-derived pigments and four treatments fortified with different natural color extracts, e.g. HB: 10% hibiscus anthocyanin, TR: 4% curcumin. SP: 6% spinach chlorophyll, and BP: 4% blue pea anthocyanin.

#### Analysis of physicochemical properties

All the physicochemical properties were tested on 1, 7, and 14 days of storage at 4 °C in triplicates. Total phenolic content (TPC) was tested on the 1st and the 14th days in triplicates.

#### pH and titratable acidity (TTA)

pH was determined using a pH meter (PH 450, thermo scientific, Singapore). Titratable acidity was measured as per Jayarathna et al. (2020). In brief, 9 mL of distilled water was added to 1 mL of yogurt with 3 drops of phenolphthalein 0.1% (w/v) and then titrated against 0.1 N NaOH.

### Syneresis

As per Arab et al. (2020) centrifugation of 10 mL of yogurt samples at 700× g for 10 min was followed. Then, the clear supernatant was weighed and expressed as a percentage of the initial weight of the yogurt sample.

### Flowability

Flowability was measured using wire mesh with a mesh size of 1.69 cm^2^. 100 mL from each yogurt sample was poured onto it and erected the mesh facilitating the flow. The area of flow was calculated, respect to the number of holes covered with yogurt.

### Sedimentation

For all the treatments, 100 mL from each was poured into bottles of 125 mL volume and air tightened using the corks. Then was kept in a stable position under refrigerated temperature (4 °C). Any changes in sedimentation and color separation were observed and captured through photographs during the entire storage of 14 days.

### Proximate composition analysis

The content of crude fat (CF), crude protein (CP), total solids (TS), and ash was measured according to methods described by Matela, Pillai, Matebesi-Ranthimo, and Ntakatsane (2019). Crude protein was determined by the Kjeldahl method (N × 6.38). Soxhlet apparatus (SER145, Italy) with petroleum ether was used in crude fat detection. Total solids were determined with respect to the moisture content. Initially, 5 g of the samples were oven-dried for 3 h at 105 °C to remove the moisture content. Then, subtracting the moisture contnt by 100, total solid content was determined. Ash content was measured by the incineration of 2 g of each sample in a muffle furnace (DMF-05, HumanLab, Korea) for 3 h at 550 °C.

### Color

Color was directly measured by the colorimeter (CR-10, Konica Minolta, Japan). L*, a*, and b* values were reported.

### Sensory analysis

The organoleptic qualities of naturally colored probiotic stirred yogurts were evaluated using 30 panelists, after a brief introduction to the sensory analysis of yogurt. The same panel was used to evaluate after 1, 7, and 14 days of storage for triplicated yogurt products. A 5-point hedonic scale from 1 for “liked very much” to 5 for “disliked very much” was given to score. The panelist was asked to evaluate the appearance, flavor, color, texture, aroma, and overall acceptance of the yogurt samples presented at room temperature.

### Total phenolic content

Total phenolic contents were detected, using Folin-Ciocalteu reagent as reported by Moldovan, Iasko, and David l (2016). Expressed as mg gallic acid equivalents (GAE)/L of yogurt.

### Microbiological analysis

After pre-preparations of the respective media, the presence of the following bacteria was tested. Samples were diluted up to 10^−6^ while the pour plate technique was practiced. Coliforms were cultured in the McConkey media and incubated for 24 h at 37℃, aerobically (Matin et al., 2018). Bifidobacteria Selective Count Agar Base was used to culture Bb12 under an incubation temperature of 37℃ for 72 h under anaerobic conditions (in anaerobic jars) (Arab et al., 2020). MRS agar plates were incubated for 48 h at 37℃ to analyze the total lactic acid bacteria (*Streptococcus thermophilus* and *Lactobacillus delbrueckii* subsp. Bulgaricus) (Hong, Son, Kwon, & Kim, 2020). Enumeration of *Streptococcus thermophilus* was carried out on M17 agar under aerobic incubation at 37℃ for 72 h (Shalaby & Amin, 2018). conditions. Yeast and mold plates were incubated for 2–5 days at 32℃ under aerobic conditions in Potato Dextrose Agar. The two strains were counted separately. Colony counts were expressed in log_10_ colony-forming units (CFU/mL).

### Statistical analysis

All statistical analyses for means were carried out using the SAS version 9.4 (SAS Institute, Cary, NC). Mean separations were done using Tukey’s HSD test. Sensory evaluation was done by Friedman test using SPSS with a significant difference of (*p* < 0.05). Principal component analysis (PCA) was used in multivariate analyses of all studied observations, using the software Simca 16.0 (Sartorius Stedim Data Analytics AB, Umeå, Sweden). The variables were pre-processed with UV centring. PCA score scatter plots were developed for assessing similarities and groupings of studied parameters, while PCA loading scatter plots were used to interpret the score scatter plots to display similarities or differences among all variables. SigmaPlot 14 (SPSS, Chicago, USA) was used in creating graphs.

## Results and discussion

### Extraction and incorporation of pigments into yogurt

Extracted anthocyanins from Hibiscus showed a purplish-red color, without any noticeable color changes for two days at 4℃. In the present study, pigments from the blue pea retained a shelf life in between 21 and 22 weeks at 4 °C. It is in agreement with Hellstrom, Mattila, and Karjalainen (2013) on the anthocyanin stability of berry juices, which were retained for 22 weeks at 4 °C. Blue-hued anthocyanins in blue pea are high-acylated delphinidin derivatives, which had the stability and anthocyanin retention of.

58% at 10 °C when extracted into distilled water by 90 days of storage (Pham, Le, Nguyen, Tran, Dao, Nguyen, & Anh, 2020). Curcumin in turmeric resulted in yellow-orange color extract, with low solubility, in agreement with Shalaby and Amin (2018). Chlorophyll, in spinach, resulted in dark green with no observable color deterioration at 4 °C, until four weeks similar to Kidmose, Edelenbos, Christensen, and Hegelund (2005).

### Physicochemical properties

**Fig. 2:**
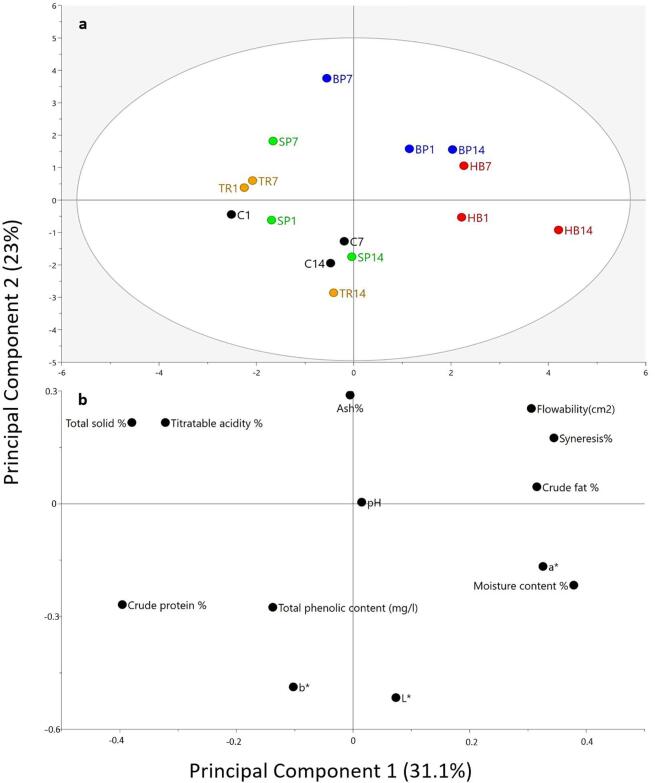
Principal component analysis of physicochemical properties of stirred yogurt (C: control) and added plant-derived colorants, i.e. HB: Hibiscus, TR: Turmeric, SP: Spinach, BP: Blue pea. (a): score plot and (b): loading plot.

PCA model, explaining 31.1 and 23 percent of the variance in the first 2 principal components, respectively, was used to evaluate the overall variation of physicochemical properties of stirred yogurt with added plant colorants, i.e. HB: Hibiscus, TR: Turmeric, SP: Spinach, BP: Blue pea. The score plot (Fig. 2A) suggested the tendency of grouping yogurt along the diagonal of top-right to bottom-left. Yogurt incorporated with hibiscus and blue pea pigments were grouped on the topright side while the rest were located on the bottom-left side. According to the loading plot (Fig. 2B), the underlying reasons for this variation could be comprehended. Flowability and syneresis are comparatively higher for yogurt produced with hibiscus and blue pea, while total solids and titratable acidity are higher in other groups. In addition, Fig. 2A shows, flowability and syneresis are positively correlated. This because of flowability is a result of higher liquid presence in the yogurt, which increases the chances of expelling to the surface, resulting in higher values for syneresis. Furthermore, total solid is negatively correlated with the moisture content.

### pH and TTA

Anthocyanin change their color with the pH, even when used in milk (Madushan, Vidanarachchi, Prasanna, Werellagama, & Priyashantha, 2021)*.* In all the studied treatments, pH value declined over the storage due to post-acidification (Table 1), irrespective of including plant-derived colorants. The occurrence of post-acidification is due to the slow-phased continuation of lactic acid production by acid-tolerant *Lactobacillus delbrueckii* subsp. *bulgaricus* during refrigerated storage (Deshwal, Tiwari, Kumar, Raman, & Kadyan, 2021). This also could affect the sensory qualities and stability of pigments (Scibisz, Ziarno, & Mitek, 2019). Variation in TTA% values with the storage is shown in Table 1. TTA% did not vary over the shelf-life considering treatments, although reduced with time, where the highest percentage was in turmeric (1.98%) followed by blue pea, control, spinach, and hibiscus (1.59%) by the end of the 14th day. This reduction of TTA over the storage is contradicted to the results of Shalaby and Amin (2018), who showed increasing TTA upon storage. TTA% did not differ between treatments on the first day after the storage, however, after 7 and 14 days of storage.

**Table 1:** Physicochemical properties and bacterial count of colored probiotic stirred yogurt at 4℃.

Parameter	ST^1^	Treatment				
		C^3^	HB^3^	TR^3^	SP^3^	BP^3^
		Mean ± SD^1^	Mean ± SD	Mean ± SD	Mean ± SD	Mean ± SD
pH	1	5.0 1 ± 0.00^Aab^	5.06 ± 0.01^Aa^	4.9 9 ± 0.02^Aab^	4.98 ± 0.02^Ab^	4.98 ± 0.02^Ab^
	7	4.95 ± 0.01^Aab^	4.99 ± 0.01^Ba^	4.9 2 ± 0.01^Bb^	4.93 ± 0.01^ABb^	4.91 ± 0.01^Bb^
	14	4.94 ± 0.04^Aa^	4.9 5 ± 0.01^Ba^	4.87 ± 0.01^Ca^	4.89 ± 0.01^Ba^	4.87 ± 0.01^Ba^
TTA^2^ (%)	1	2.73 ± 0.08^Aa^	2.01 ± 0.45^Aa^	2.3 1 ± 0.06^Aa^	2.1 2 ± 0.05^Aa^	2.40 ± 0.26^Aa^
	7	2.31 ± 0.06^Ba^	1.83 ± 0.30^Aa^	2.25 ± 0.05^Aa^	2.225 ± 0.10^Aa^	2.37 ± 0.03^Aa^
	14	1.74 ± 0.13^Ca^	1.59 ± 0.06^Aa^	1.98 ± 0.16^Aa^	1.6 2 ± 0.16^Ba^	1.80 ± 0.10^Aa^
Syn^2^ (%)	1	18.82 ± 3.96^Ab^	30.69 ± 3.20^Aa^	32.53 ± 0.73^Aa^	29.02 ± 0.58^Aab^	36.18 ± 0.88^Aa^
	7	24.16 ± 2.19^Ab^	36.86 ± 1.79^Aa^	29.79 ± 1.88^Aab^	30.89 ± 2.27^Aab^	33.24 ± 1.50^Aa^
	14	29.02 ± 2.36^Ac^	39.20 ± 0.39^Aa^	32.03 ± 0.96^Abc^	32.15 ± 1.80^Abc^	36.44 ± 0.31^Aab^
Flow^2^ (%)	1	711.80 ± 42.38^Ab^	834.3 0 ± 19.03^Ab^	755.71 ± 18.08^Ab^	713.40 ± 20.22^Ab^	1018.5 1 ± 25.09^Aa^
	7	769.77 ± 31.47^Ab^	1236.10 ± 29.03^Aa^	804.16 ± 21.05^Ab^	1189.48 ± 6.10^Aa^	1158.50 ± 36.09^Aa^
	14	788.10 ± 15.62^Ab^	1276.23 ± 4.40^Ba^	814.86 ± 3.66^Ab^	1203.56 ± 7.93^Ba^	1182.16 ± 6.56^Ba^
CP^2^ (%)	1	3.04 ± 0.02^Aa^	2.61 ± 0.06^Ab^	2.95 ± 0.04^Aa^	2.94 ± 0.03^Aa^	2.70 ± 0.03^Ab^
	7	3.03 ± 0.02^Aa^	2.68 ± 0.03^Ab^	2.94 ± 0.07^Aa^	2.98 ± 0.02^Aa^	2.70 ± 0.04^Ab^
	14	3.00 ± 0.04^Aa^	2.6 8 ± 0.02^Ab^	2.94 ± 0.04^Aa^	2.99 ± 0.04^Aa^	2.71 ± 0.02^Ab^
CF^2^ (%)	1	4.00 ± 0.48^Aab^	5.44 ± 0.01^Aa^	4.28 ± 0.57^Aa^	2.62 ± 0.18^Ab^	4.13 ± 0.13^Aab^
	7	3.96 ± 0.94^Aa^	5.47 ± 0.80^Aa^	4.21 ± 1.53^Aa^	2.64 ± 0.84^Aa^	4.10 ± 0.08^Aa^
	14	3.42 ± 1.65^Aa^	5.36 ± 1.81^Aa^	4.26 ± 0.72^Aa^	2.63 ± 0.79^Aa^	4.09 ± 0.49^Aa^
MC^2^ (%)	1	79.60 ± 0.07^Aa^	80.86 ± 0.08^Aa^	78.94 ± 1.59^Aa^	79.89 ± 0.09^Aa^	80.87 ± 0.08^Aa^
	7	81.39 ± 1.86^Aa^	80.23 ± 0.39^Aa^	79.25 ± 0.25^Aa^	79.28 ± 0.41^Aa^	79.12 ± 0.06^Aa^
	14	80.07 ± 0.21^Aa^	81.22 ± 0.41^Aa^	80.65 ± 0.19^Aa^	80.21 ± 0.08^Aa^	81.13 ± 0.31^Aa^
TS^2^ (%)	1	20.40 ± 0.0^Aa^	19.14 ± 0.08^Aa^	21.06 ± 1.59^Aa^	20.1 1 ± 0.09^Aa^	19.13 ± 0.08^Aa^
	7	18.61 ± 1.86^Aa^	19.77 ± 0.39^Aa^	20.75 ± 0.25^Aa^	20.72 ± 0.41^Aa^	20.88 ± 0.06^Ba^
	14	19.93 ± 0.21^Aa^	18.78 ± 0.41^Aa^	19.35 ± 0.19^Aa^	19.79 ± 0.08^Aa^	18.87 ± 0.31^Ba^
Ash (%)	1	0.65 ± 0.07^Aa^	0.54 ± 0.06^Aa^	0.70 ± 0.01^Aa^	0.69 ± 0.02^Aa^	0.61 ± 0.16^Aa^
	7	0.80 ± 0.02^Aa^	0.54 ± 0.09^Aa^	0.71 ± 0.03^Aa^	0.67 ± 0.17^Aa^	1.52 ± 0.78^Aa^
	14	0.80 ± 0.03^Aa^	0.54 ± 0.10^Aa^	0.73 ± 0.02^Aa^	0.70 ± 0.10^Aa^	1.58 ± 0.83^Aa^
TPC 2	1	42.75 ± 0.74^Bc^	54.29 ± 1.99^Abc^	60.09 ± 3.93^Aab^	69.14 ± 4.59^Aa^	46.49 ± 1.08^Bc^
	14	54.58 ± 0.81^Ab^	47.86 ± 6.40^Ab^	72.64 ± 2.82^Aa^	63.23 ± 2.28^Aab^	54.84 ± 0.62^Ab^
*St*^2^	1	14.69 ± 0.03^Aa^	14.00 ± 0.00^Ac^	12.48 ± 0.05^Ad^	12.52 ± 0.07^Ad^	14.32 ± 0.05^Ab^
	14	11.88 ± 0.15^Ba^	10.62 ± 0.04^Bb^	9.81 ± 0.04^Bc^	9.77 ± 0.10^Bc^	9.53 ± 0.08^Bc^
TLAB^2^	1	14.70 ± 0.07^Ac^	15.06 ± 0.03^Ab^	13.03 ± 0.07^Bd^	11.36 ± 0.03^Be^	15.66 ± 0.02^Ba^
	14	13.94 ± 0.10^Ba^	13.77 ± 0.20^Ba^	13.69 ± 0.10^Aa^	14.90 ± 0.01^Aa^	13.99 ± 0.00^Aa^
Bb12^2^	1	14.58 ± 0.07^Aa^	13.38 ± 0.12^Ac^	13.62 ± 0.21^Abc^	14.19 ± 0.02^Aab^	13.77 ± 0.24^Abc^
	14	13.77 ± 0.0-^Ba^	13.73 ± 0.00^Aa^	9.81 ± 0.04^Bd^	13.00 ± 0.00^Bb^	10.82 ± 0.09^Bc^
E-coli	1	0.00 ± 0.00^Aa^	1.00 ± 1.41^Aa^	0.00 ± 0.00^Aa^	1.00 ± 1.41^Aa^	0.00 ± 0.00^Ba^
	14	1.00 ± 1.41^Aa^	2.05 ± 0.00^Aa^	0.00 ± 0.00^Aa^	2.10 ± 0.21^Aa^	2.00 ± 0.00^Aa^
Yeast	1	0.00 ± 0.00^Aa^	0.00 ± 0.00^Ba^	0.00 ± 0.00^Aa^	0.00 ± 0.00^Aa^	0.00 ± 0.00^Ba^
	14	1.15 ± 1.62^Aa^	2.00 ± 0.00^Aa^	1.15 ± 1.62^Aa^	0.00 ± 0.00^Aa^	2.23 ± 0.33^Aa^
Mold	1	0.00 ± 0.00^Aa^	0.00 ± 0.00^Ba^	2.00 ± 0.00^Aa^	0.00 ± 0.00^Ba^	0.00 ± 0.00^Aa^
	14	1.00 ± 1.41^Aa^	0.00 ± 0.00^Aa^	2.20 ± 0.30^Aa^	2.00 ± 0.00^Aa^	1.10 ± 1.60^Aa^

Mean values with average of triplicates are shown.

1 SD: Standard Deviation; ST: Storage Day.

2 TTA: Titratable acidity%; Syn: syneresis; Flow: Flowability; CP: crude protein; CF: crude fat; MC: moisture content; TS: total solids; TPC: total phenolic content mg gallic acid equivalents (GAE)/L of yogurt, *St*: *Streptococcus thermophilus;* TLAB: Total Lactic Acid Bacteria; Bb12: *Bifidobacterium bifidum*. Microbiological analysis are in log 10 CFU/mL.

3 C: Control; TR: Turmeric; SP: Spinach; BP: Blue pea. Means with different uppercase superscript are significantly different (*P* < 0.05) within the same parameter for three different storage days. Means with different lowercase superscript are significantly different (*p* < 0.05) within the same row for different treatments.

### Syneresis

Accumulation of whey on the gel surface leads to poor consumer acceptance. Senadeera et al. (2018) observed that incorporation of *Annona* fruit pulps resulted in greater syneresis compared to the control in agreement with the observations in the present study. The basis of syneresis in all the treatments including the control is due to the reduction of pH and subsequent contraction of the casein network facilitating the expulsion of whey onto the surface. Syneresis values significantly varied between treatments (Table 1), while values for control always tend to be lower compared to the colorant added yogurts. This observation is likely to relate to the thermodynamic incompatibility between polysaccharides of plant materials and milk proteins as discussed by Senadeera et al. (2018). Syneresis of colored yoghurt was higher than the control as the aqueous pigment extractions were added to the yoghurt after fermentation. This is in agreement with Vareltzis, Adamopoulos, Stavrakakis, Stefanakis, and Goula (2016) who tested the hypothesis that extra moisture can be retained in stirred yoghurt-based products by introducing the extra moisture before the reformation of yoghurt.

### Flowability

On the first day of storage blue pea incorporated yogurt showed the highest flowability (Table 1). This since the higher solvent (water) ratio (1:20) (g/mL) used for the extraction of blue pea pigments compared to the rest and as observed of highest moisture content (Table 1) on the first day. The flowability of hibiscus, spinach, and blue pea incorporated probiotic yogurts resulted in increased flowability at the end of storage compared to the first and seventh day of storage. This most likely due to weakened intermolecular bonds between proteins and plant materials over the storage period.

### Sedimentation

**Fig. 3:**
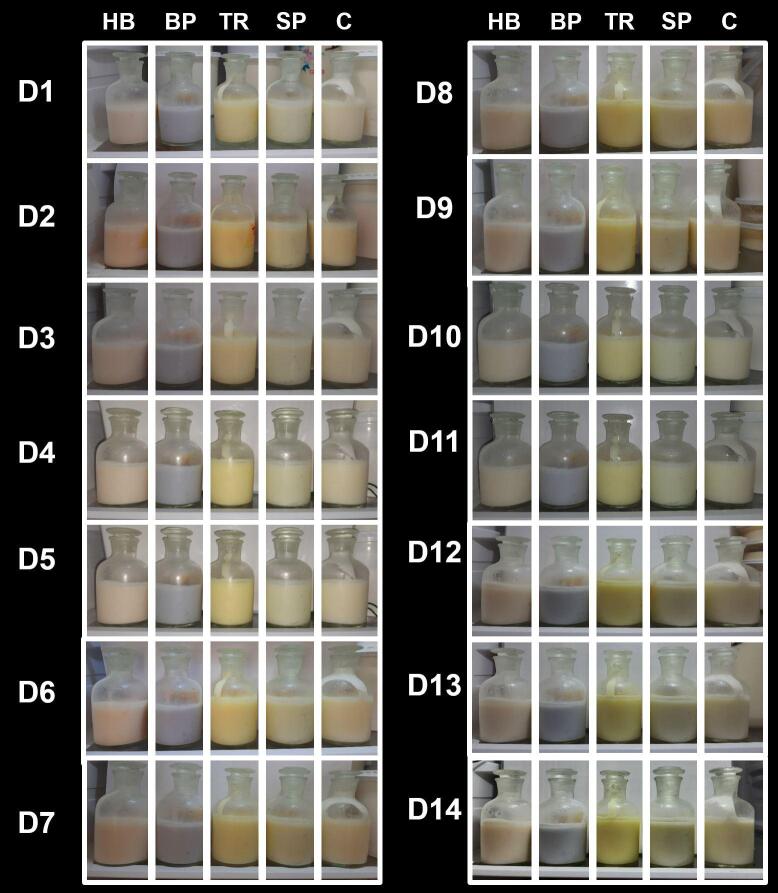
Sedimentation of colored probiotic stirred yogurt at 4℃ within the storage of 14 days. Always stirred yogurts were kept in the order of control: D1-D14 are storage days from day 1 to day 14. C: Control yogurt without colorants, HB: Hibiscus, TR: Turmeric, SP: Spinach, BP: Blue pea.

No visible color changes, phase separation, or sedimentations were observed throughout the 14 days of storage under 4 °C as shown in Fig. 3. Phase separation of stirred yogurt is undesirable and therefore, our study shows the incorporation of plant materials in the specified concentration would not lead to unwanted sedimentation during the storage. Thus, products are likely to have optimal final physical quality until the end of shelf-life. In yogurt production, mostly stabilizers are used to avoid this phenomenon of phase separation, especially the use of polysaccharides that has gained much attention (Everett & McLeod, 2005). Yet, in the present study, produced yogurts did not sediment or phase-separated, illustrating the potential stabilizing effect of the added plant colorants.

### Proximate analysis

CP ranges from 2.6% to 3.0% (Table 1), within the acceptable levels as specified by the Codex Alimentarius (2003) and in agreement with Shalaby and Amin (2018). All the colored yogurts have shown lower CP values than the control. CF of spinach has shown a lower significant value on the first day compared to the other treatments (Table 1). Afterward, no significant differences among the treatments in terms of CF were detected. Moreover, these values comply with the standard of having <15% CF (Codex Alimentarius (2003)).

The moisture content of the present study is in agreement with previous findings of Pires et al. (2018) who showed edible flower incorporated yogurts ranged 84–85% of moisture content. Total solids had no significant differences between the treatments. Ash content was ranged from 0.5%1.5% and was insignificant in both, day and treatment wise. However, in contrast to this study, cabbage anthocyanin (10%) and curcumin (10%) (Shalaby & Amin, 2018) incorporated yogurts have resulted in significantly different ash contents in between. Different derivatives of anthocyanin and lower concentration (4%) of curcumin in the present study might explain the discrepancies between the results of these studies.

### Color

**Fig. 4:**
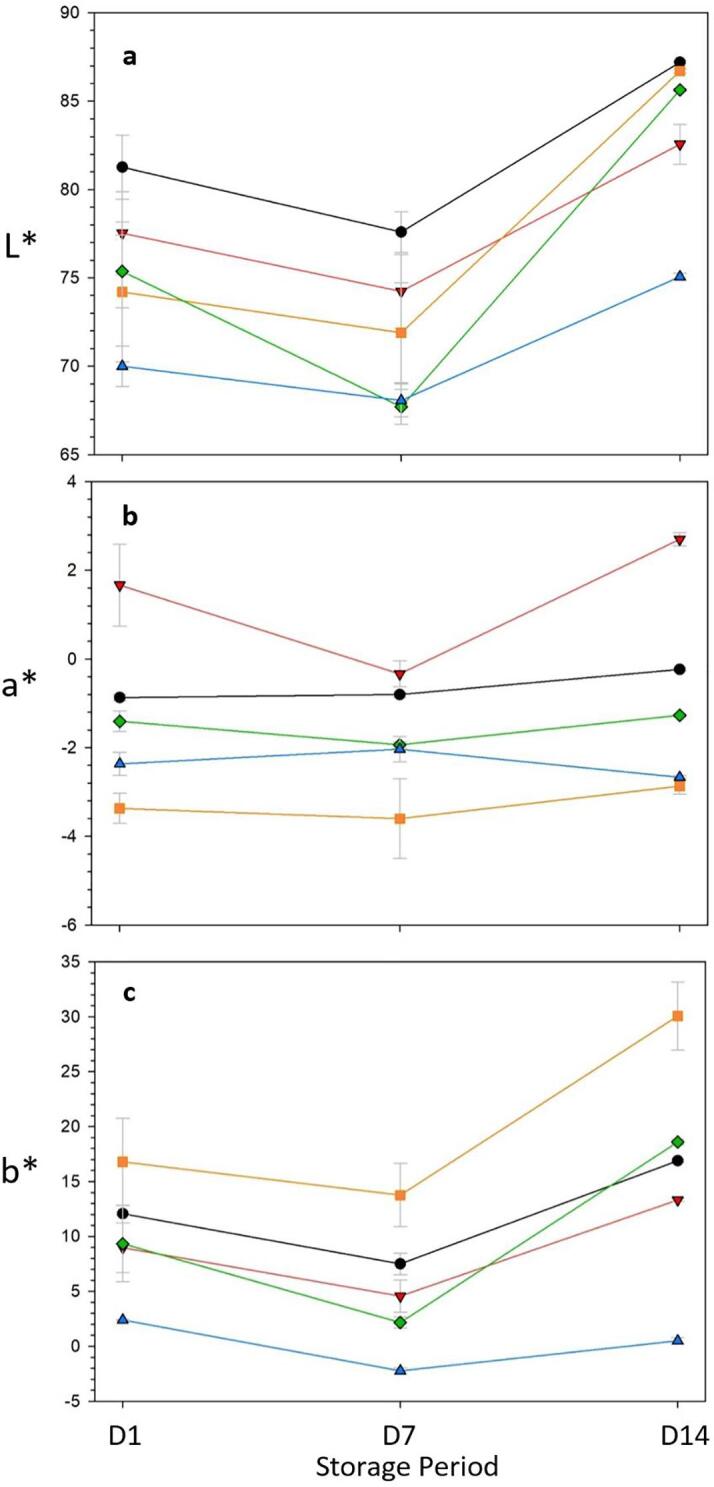
L* (a), a*(b), b* (c) values of probiotic stirred yogurt (Black: control) and added plant-derived colorants, i.e. Red: Hibiscus, Orange: Turmeric, Green: Spinach, Blue: Blue pea over the 14 days of storage D1, D7 and D14 are one, seven and fourteen days after the storage, respectively.

Color is one of the crucial physicochemical properties evaluated in the present study. The study, aimed at evaluating the stability of the color over the storage by measuring L*, a*, and b* values. The incorporation of plant materials as colorants reduced the lightness (L*) compared to the control sample (Fig. 4A). Blue pea incorporated stirred yogurt resulted in the lowest L* on the first and last day of storage. L* values were generally increased at the end of the storage for all treatments, although on the 7th-day values are lowered compared to the first day. This increase of L* indicates possible degradation or oxidation of the color pigments and therefore lightness of the colorant is increased. This is in agreement with several studies such as red rice pigmented yogurt (Chen, Lv, Du, & Chen, 2012) and fruity yogurt (Pires et al., 2018). In contrast to our observations, grape incorporated kefir (Montibeller et al., 2018), which showed a decrease in L* value over time. Pires et al. (2018) suggested yogurt starter cultures could cause an adverse impact on the stability of anthocyanin by accelerating their destruction through enzymes like glycosidase. Further, high losses of anthocyanin can also occur due to the presence of higher oxygen concentrations incorporated during the stirring of fermented gel. As expected a* (red/green) value is higher in hibiscus incorporated yogurt (Fig. 4B) while b* (yellow/blue) value is higher in turmeric (Fig. 4C). Yellow color intensity was increased in turmeric-incorporated yogurt on the 14th day than the 1st day. Although, measured color values resulted in changes over storage no visible color change was detected.

### Sensory evaluation

**Fig. 5:**
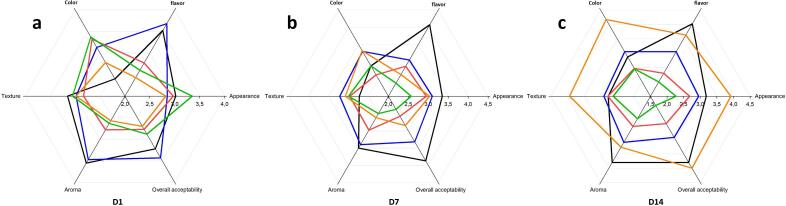
Sensory evaluation probiotic stirred yogurt (Black: control) and added plant-derived colorants, i.e. Red: Hibiscus, Orange: Turmeric, Green: Spinach, Blue: Blue pea over the 14 days of storage D1, D7 and D14 are one (a), seven (b) and fourteen (c) days after the storage, respectively.

The same 30 sensory panelists used in preliminary screening were used in this sensory evaluation to score the same attributes, color, texture, flavor, aroma, appearance, and overall acceptability in each product on days 1, 7 and 14 of storage, including the control. Instead of choosing the best-preferred yoghurt, we checked for the mean consumer liking score separately from the product with respect to attributes. Therefore, each attribute on each day had differently preferred products. The added plant pigments probably negatively influenced the aroma of the treated samples. On the first day, the turmeric aroma was much stronger giving it the lowest score while on the 7th and 14th spinach yogurt became the least preferred (Fig. 5). Depending on results, it is suggested to use a different solvent instead of ethanol to extract spinach. The appearance was most preferred in the spinach yogurt on the first day of storage while on the same day least preference was ranked for turmeric yogurt. However, surprisingly on the 14th day, a highly accepted appearance was with the turmeric yogurt, followed by control, blue pea, hibiscus, and spinach. Except for day 1 rest of the two days, control has scored as the most flavored. The highest mean consumer liking score for the flavor of non-colored yogurt compared to colored yogurts (Jabuticaba and Jamelao peel) by Freitas-Sa et al. (2018) is in parallel to the above result. The flavor would need to live up to the consumers’ expectations delivered by the color.

The color of the control was the least preferred on day 1 (Fig. 5A). Turmeric which ranked the 4th favored color on the first day became the most favorable color afterwards (on both 7th and 14th). Consistently, Shalaby and Amin (2018) have represented that the stirred yogurt with 10% of turmeric has scored a lower point than the same amount of curcumin though both of them were accepted by the consumers. Considering, overall acceptability, the highest was with blue pea followed by control, spinach, hibiscus, and turmeric on the first day of storage. When it comes to the end of the storage, superior quality was observed in turmeric-incorporated yogurt. On day 1 and 7, the appearance, texture, and overall acceptability were not significantly different (*p* > 0.05). However, all of the evaluated attributes showed significance on the 14th day. In agreement, Chen et al. (2012) have also, gain insignificant appearance and texture in their red rice pigmented yogurt at 2 weeks under 4 °C.

### Total phenolic content analysis

The TPC values obtained in the control are due to the presence of polyphenols in milk, which are derived from protein, feed, and reducing compounds (Senadeera et al., 2018). On the first day of the storage the highest (69.1 mg GAE/L) and lowest (42.7 mg GAE/L) TPC were observed in spinach and control, respectively. Also, all the colored yogurts showed higher TPC values than the control on the day one. Similarly, yogurts supplemented with grape and callus extracts reported higher TPC (Karaaslan, Ozden, Vardin, & Turkoglu, 2011). However, on the 14th day, turmeric incorporation resulted in the highest TPC (72.6 mg GAE/L) while spinach ranked second. Control, hibiscus and blue pea were not significantly different on the last day of storage. Over the storage every product was with a TPC value of more than 42.7 mg GAE/L. The highest (69.1 mg GAE/L) and lowest (42.7 mg GAE/L) TPC were observed in spinach and control, respectively at the beginning of the storage. Also, all the colored yogurts showed higher TPC values than the control on the 1st day. Similarly, yogurts supplemented with grape and callus extracts reported higher TPC (Karaaslan et al., 2011). However, on the 14th day, turmeric incorporation resulted in the highest TPC (72.6 mg GAE/L). According to Shalaby and Amin (2018), who demonstrated a linear relationship between TPC and antioxidant capacity of colored yogurts, we can also speculate the turmeric-incorporated yogurt might have higher antioxidants, which can deliver beneficial health effects.

### Microbiological quality

Table 1 represents the variation of bacterial counts of starter cultures, probiotics, E-coli, yeast, and mold respectively. Starter culture count among the treatments has shown significant differences due to the acidification of yogurt or the presence of ascorbic acid in plant extracts. Scibisz et al. (2019) stated ascorbic acid reduces the amount of oxygen required for the viability of *S. thermophilus* in blended yogurt. Further, they confirmed all the treatments showed an acceptable starter culture count. This is consistent with our data of observing counts greater than 7 log CFU/mL. Fresh yogurts have shown a total lactic acid bacteria count varied from 11.3 to 15.0 log_10_ CFU/mL. Then, it has declined to 13.7–14.9 log _10_ CFU/mL by the 14th day. Furthermore, Shalaby and Amin (2018) have stated that turmeric has shown antimicrobial properties and therefore adversely affects the growth of starter culture while anthocyanin facilitates the metabolic rate and growth of starter cultures. Comparing the results gained for turmeric, blue pea and hibiscus on the first day, it is clear that the findings support the above fact (on day 1, HB: 15.0 log_10_ CFU/mL, TR: 13.0 log_10_ CFU/mL, BP: 15.6 log_10_ CFU/mL). Probiotic counts even after 14 days of storage in all treatments have shown over 6 logs CFU/mL that is accepted (Codex Alimentarius (2003)). In addition, there were significant differences in probiotic counts between the treatments. Yoghurt showed the highest significant difference to HB on day 1 while on the 14th TR had a highly varied probiotic count compared to C and HB. Obtained results show the viability loss/reduction in probiotic bacteria over the storage and that may be due to the post-acidification during the period of storage. Anyhow, the maintenance of approvable probiotic levels would be due to the content of dietary fibers within plant materials that are considered to improve their viability (Senadeera et al., 2018). Counts of E-coli, yeast, and mold were minimal or absent (Table 4).

## Conclusion

Present study provides simultaneous comparisons of natural plant pigments and physicochemical properties, in fermented milk. The results demonstrated that the manufacturing of colored stirred yogurts with studied natural pigments is not creating an adverse defect on the product’s sensory perception. Simultaneously, do not exert an adverse effect on the total lactic acid bacteria or probiotic bacterial count, with possibilities of maintaining bacterial counts above the required limits. In addition, an enhancement of TPC values in colored yogurts was affirmed along with a higher sensory acceptance. Findings and insights from this study will contribute to comprehensive technical and technological applications for fermented milk. Further research into the choice of natural pigments, health effects, and technologies for pigment extraction will increase the industrial applicability of the study. Moreover, research is guaranteed to investigate the efficient extraction strategies of the pigments from those plant sources to upscale the production of yogurt. Additionally, studies focus on nutritional and health benefits are recommended to confirm the potentials of delivering health-promoting benefits of studied plant materials.

## Declaration of Competing Interest

The authors declare that they have no known competing financial interests or personal relationships that could have appeared to influence the work reported in this paper.
